# Differentiated and Poorly Differentiated Thyroid Carcinoma in Ovarian Teratoma With Primary Papillary Thyroid Carcinoma: A Series of Two Case Reports

**DOI:** 10.7759/cureus.104169

**Published:** 2026-02-24

**Authors:** Tanveer Fatima, Saffa Ilyas, Aliza Hameed, Waqas Shafiq, Ahmed Imran Siddiqi, Nida Babar, Ammara Yasmin, Midhat Waheed, Mudassar Hussain

**Affiliations:** 1 Endocrinology and Diabetes, Shaukat Khanum Memorial Cancer Hospital and Research Centre, Lahore, PAK; 2 Endocrinology, Diabetes and Metabolism, Shaukat Khanum Memorial Cancer Hospital and Research Centre, Lahore, PAK; 3 Internal Medicine, Shaukat Khanum Memorial Cancer Hospital and Research Centre, Lahore, PAK; 4 Pathology, Shaukat Khanum Memorial Cancer Hospital and Research Centre, Lahore, PAK; 5 Oncology, Shaukat Khanum Memorial Cancer Hospital and Research Centre, Lahore, PAK; 6 Histopathology, Shaukat Khanum Memorial Cancer Hospital and Research Centre, Lahore, PAK

**Keywords:** molecular testing in thyroid cancer, multidisciplinary team, papillary thyroid carcinoma in ovarian teratoma, poorly differentiated thyroid carcinoma in ovarian teratoma, teratoma, thyroid cancer in ovarian teratoma

## Abstract

Teratoma is a tumor that originates from one or more layers of germ cells. It may contain several types of tissues. Ovarian teratomas are the most common amongst all teratomas. These can be immature, mature, and monodermal. Incidence of thyroid carcinoma originating from ovarian teratoma is rare, but cases have been reported previously.

In this case series, we describe two different cases, both presenting at separate times during their treatment of thyroid carcinoma in ovarian teratoma.

The first patient presented to the gynecological surgery outpatient department with a history of abdominal pain and excessive menstrual bleeding. She underwent hysterectomy and oophorectomy; review of the histopathology of the specimen turned out to be ovarian teratoma with papillary thyroid carcinoma. Further workup revealed that she had a B-Raf proto-oncogene, serine/threonine kinase (BRAF) mutation. After discussing the case in a multidisciplinary team meeting, she underwent treatment for thyroidectomy completion surgery, followed by radioactive iodine therapy. Currently, she is under follow-up with the endocrinology team and disease-free.

Our second patient is an extremely rare case of concurrent presence of poorly differentiated thyroid carcinoma in ovarian teratoma with primary papillary thyroid carcinoma. She presented with thyroid swelling, which gradually increased in size to the point she developed dyspneic symptoms. She underwent total thyroidectomy for symptomatic control, and in her histopathology report, it turned out to be high-risk papillary thyroid carcinoma. She then received radioactive iodine therapy. The post-therapy scan showed uptake in thyroidectomy bed remnant thyroid tissue and intense uptake in adnexa. In the multidisciplinary team meeting, it was decided to remove the adnexal mass. Histopathology revealed poorly differentiated thyroid cancer in ovarian teratoma. She is currently on endocrinology follow-up and disease-free.

This case series highlights the rare occurrence of malignant transformation, specifically thyroid carcinoma within ovarian teratoma, and underscores the importance of thorough histological evaluation in patients with atypical presentations and relevant genetic mutations. These patients showed speedy recovery and are on follow-up with minimal post-treatment complaints, demonstrating a favorable outcome with appropriate surgical and adjuvant management.

## Introduction

Thyroid tissue originates from the endoderm. The incidence of thyroid tissue in ovarian teratoma is 5-10% [1]. Struma ovarii is a monoderm type of teratoma comprising >50% of thyroid tissue. It comprises less than 2.7% of all teratomas [2]. Malignant transformation in mature teratoma is rare (2%), with squamous cell carcinoma being the most common amongst all (75%), followed by adenocarcinoma and sarcoma [3]. It is extremely rare that thyroid tissue in ovarian teratoma transforms into differentiated thyroid carcinoma with a mere incidence of 0.1-0.3%. Papillary thyroid carcinoma, like its actual incidence in thyroid malignancies, is the most common form of thyroid cancer found in approximately 50% of ovarian teratomas. Incidence of other types of differentiated thyroid cancers in descending order includes follicular thyroid carcinoma, follicular variant of papillary thyroid carcinoma, papillary and follicular mixed thyroid carcinoma, anaplastic carcinoma, and medullary thyroid carcinoma [4].

The precise pathogenesis of thyroid carcinoma arising in teratoma is not well known due to very few reported cases; however, several hypotheses include origin from germ cells, presence of embryonic thyroid tissue remnants, somatic mutation, hormonal factors, or immune system dysfunction within the teratoma microenvironment [5].

These are typically found incidentally in histopathology. However, some patients presented with symptoms related to ovarian mass, i.e., palpable abdominal mass, abdominal pain, and dysfunctional vaginal bleeding [6].

Although imaging modalities, such as computed tomography and ultrasound, can suggest the presence of mature cystic teratoma, the identification of malignant components requires postoperative histopathology evaluation. The histological features of papillary thyroid carcinoma in ovarian teratoma are like those observed in the thyroid gland [7].

Rarely, thyroid carcinoma within a mature cystic teratoma is associated with hyperthyroidism/Graves' disease [8].

## Case presentation

Case 1 

**Figure 1:**
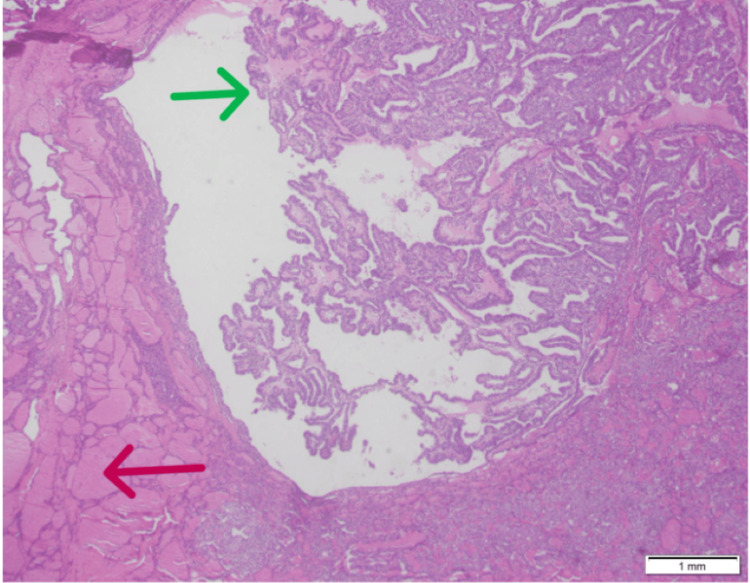
Case 1: histopathology of the ovary (red arrow: mature component of ovarian teratoma; green arrow: papillary thyroid carcinoma)

This is the case of a 42-year-old woman with a strong family history of multiple carcinomas (grandmother had colon, breast, and pancreatic cancer, and grandfather had prostate cancer). She was on a gynecologist follow-up for benign fibrocystic breast disease. She presented with abdominal pain, prompting further evaluation. Initial imaging showed a left-sided complex ovarian cyst for which she underwent left-sided salpingo-oophorectomy. The histopathology of the left fallopian tube and left ovarian cyst demonstrated "papillary thyroid carcinoma arising in teratoma" (Figure 1).

Subsequent genetic analysis showed an abnormal clone with B-Raf proto-oncogene, serine/threonine kinase (BRAF), elastase, neutrophil expressed (ELANE), casitas B-lineage lymphoma (CBL), Fanconi anemia, complementation group F (FANCF), zinc finger protein 217 (ZNF217), and programmed cell death 1 ligand 2 (PDCD1LG2) mutations. Tumor mutation burden was low: 2 mut/Mub with no evidence of microsatellite instability. 

The patient was referred to an endocrinology outpatient clinic for further management. On physical examination, she appeared clinically well with no palpable thyroid nodule. 

Initial blood workup is presented in Table 1.

**Table 1:** Case 1: biochemical analysis TSH: thyroid-stimulating hormone; hCG: human chorionic gonadotropin; AFP: alpha-fetoprotein; CA-125: cancer antigen 125; Free T4: free thyroxine; Free T3: free triiodothyronine

Investigation	Results	Normal values
Free T4	1.03	0.8-2.7 ng/dL
TSH	2.333	0.4-4.2 µIU/mL
Free T3	3.23	2.3-4.2 pg/mL
Beta-hCG, quantitative	<2	Negative <10 mIU/mL; positive =>10 mIU/mL
AFP	2.6	Adult: <8.5 ng/mL
CA-125	20.8	<35 U/mL
Calcium, corrected	8.78	8.5-10.5 mg/dL

**Figure 2:**
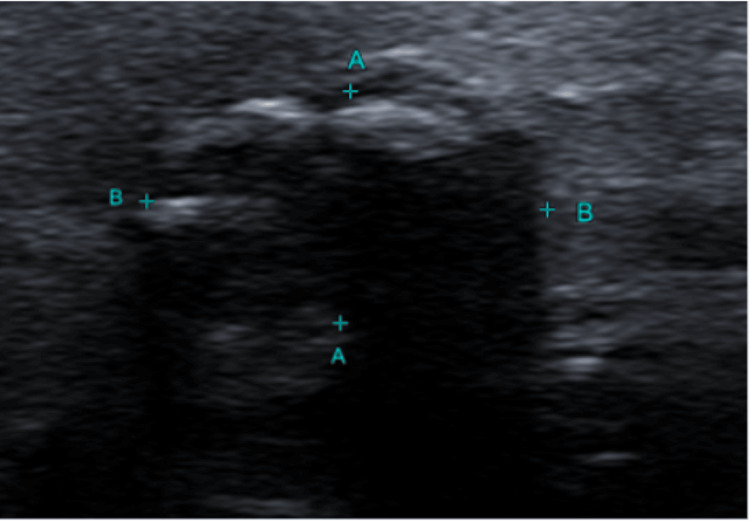
Case 1: thyroid ultrasound showing left thyroid lobe nodule (TIRADS 4) (distance A: 7.1 mm; distance B: 12.2 mm) TIRADS 4: Thyroid Imaging Reporting and Data System Category 4

Her thyroid ultrasound documented a right thyroid lobe hypoechoic solid nodule measuring 4 × 6 mm (Thyroid Imaging Reporting and Data System Category 4 (TIRADS 4)), and the left lobe showed a calcified thyroid nodule measuring 7 × 9 × 12 mm (TIRADS 4) (Figure 2).

Cytopathology of ultrasound-guided fine-needle aspiration of the left lobe TIRADS 4 nodule turned out to be a follicular neoplasm, Bethesda category IV.

**Figure 3:**
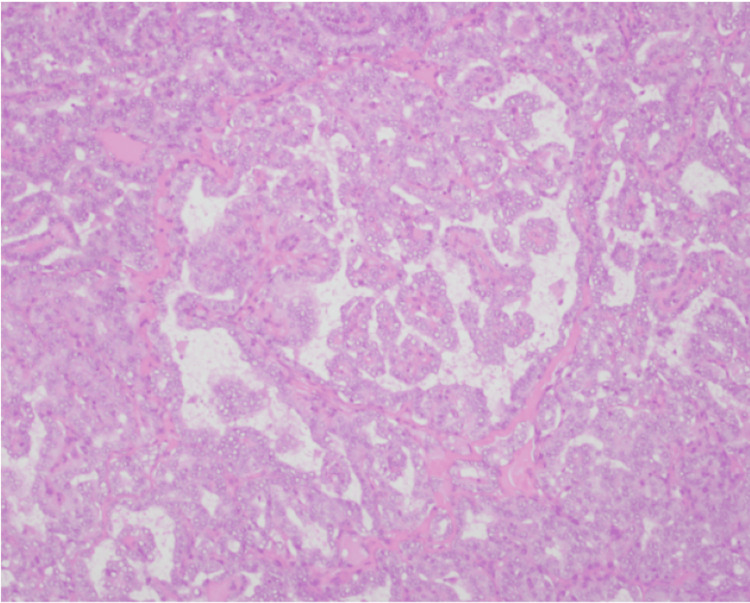
Case 1: histopathology of the thyroid specimen (papillary thyroid carcinoma)

A multidisciplinary team meeting involving endocrinologists, pathologists, surgeons, and the nuclear medicine team was convened. The team discussed that postoperative surveillance would be challenging in this patient with papillary thyroid carcinoma in ovarian teratoma with thyroid gland in situ as papillary thyroid carcinoma tumor markers will be unreliable. Given the presence of high-risk mutations, the consensus was to proceed with total thyroidectomy followed by radioactive iodine therapy. Total thyroidectomy with intraoperative findings of a 2 cm nodule in the left lobe of the thyroid was performed. Histopathology of thyroid tissue revealed "classic papillary thyroid carcinoma pT1b, pN0, pMx" (Figure 3).

**Figure 4:**
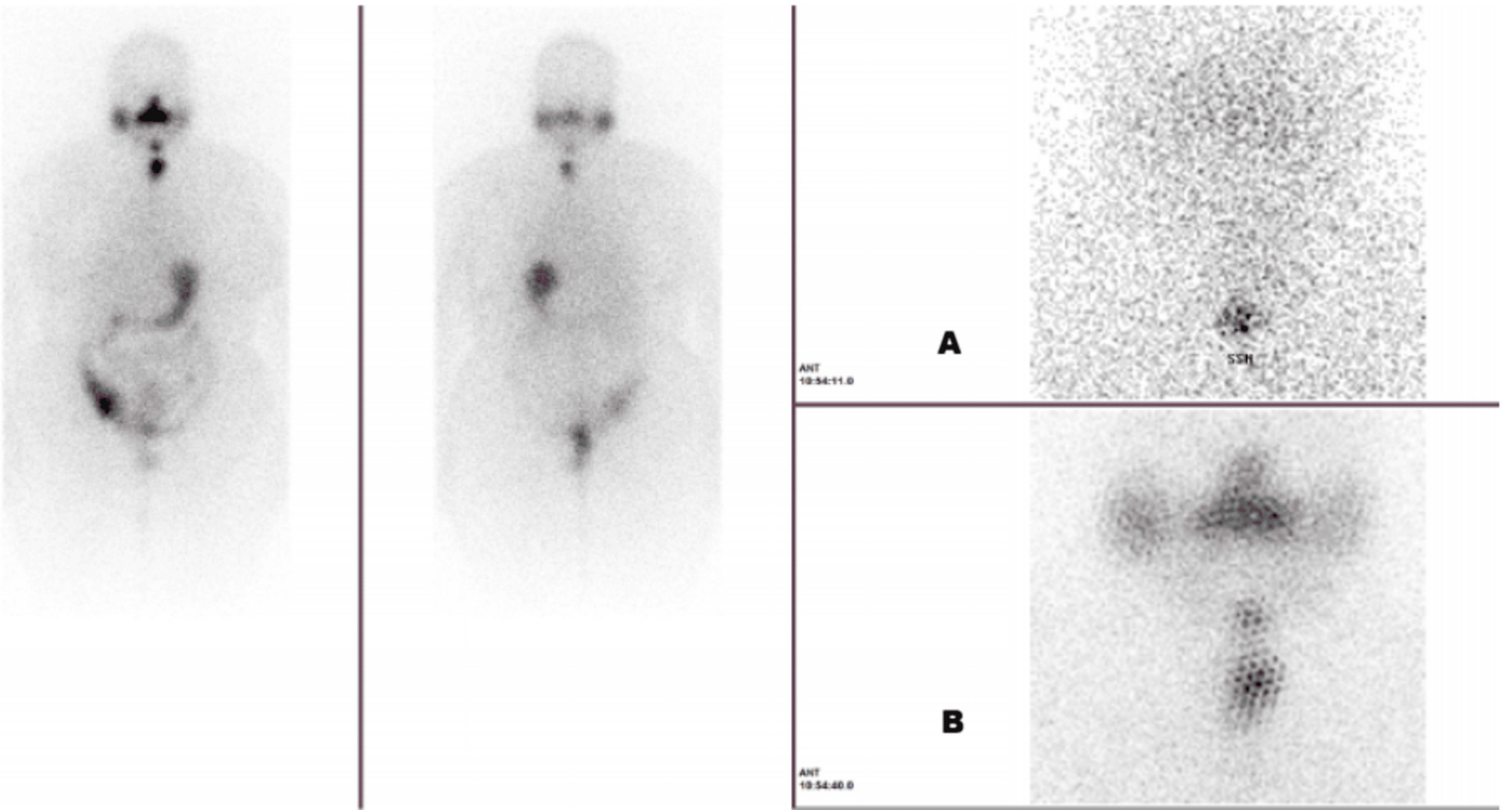
Case 1: post-radioactive iodine therapy whole body scan ((A) iodine uptake on thyroidectomy bed and (B) iodine uptake in remnant thyroid tissue and thyroglossal duct remnant)

Afterwards, she received radioactive iodine therapy. Her stimulated thyroglobulin was 48.352 ng/mL (normal athyroidic patient: <5 ng/mL). The post-therapy whole body scan report narrated that there was a well-delivered dose of Iodine-131 to the left thyroid remnant and thyroglossal duct remnant. No other radioiodine avid site was appreciated (Figure 4).

This patient is currently in remission and remains under ongoing follow-up with the endocrinology team.

Case 2

Our second case was a 34-year-old woman, with no significant family history of any malignancy or thyroid illness. She presented with progressively enlarging swelling in the anterior neck to a point that she started to have significant compressive symptoms. Her initial workup, at a local hospital, was significant for a thyroid scintigraphy scan, which demonstrated a cold nodule in the right lobe of the thyroid.

**Figure 5:**
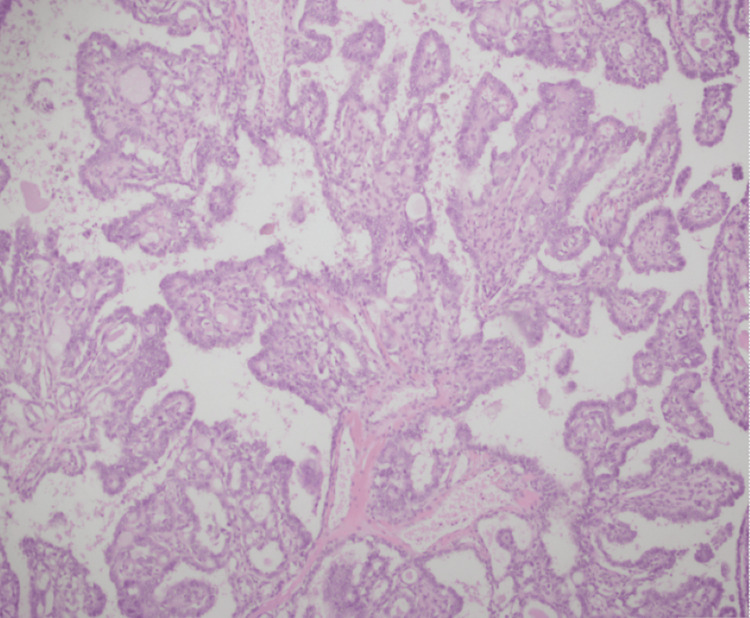
Case 2: histopathology of the thyroid specimen (papillary thyroid carcinoma)

Based on these findings, she underwent total thyroidectomy. The histopathology report of the thyroidectomy specimen was "classic papillary thyroid carcinoma, 4.7 cm, pT3aNxMx" (Figure 5).

As this was a case of high-risk papillary thyroid carcinoma based on the size of the tumor, in a multidisciplinary team meeting, it was decided that she would need radioactive iodine ablation as an adjuvant treatment.

**Figure 6:**
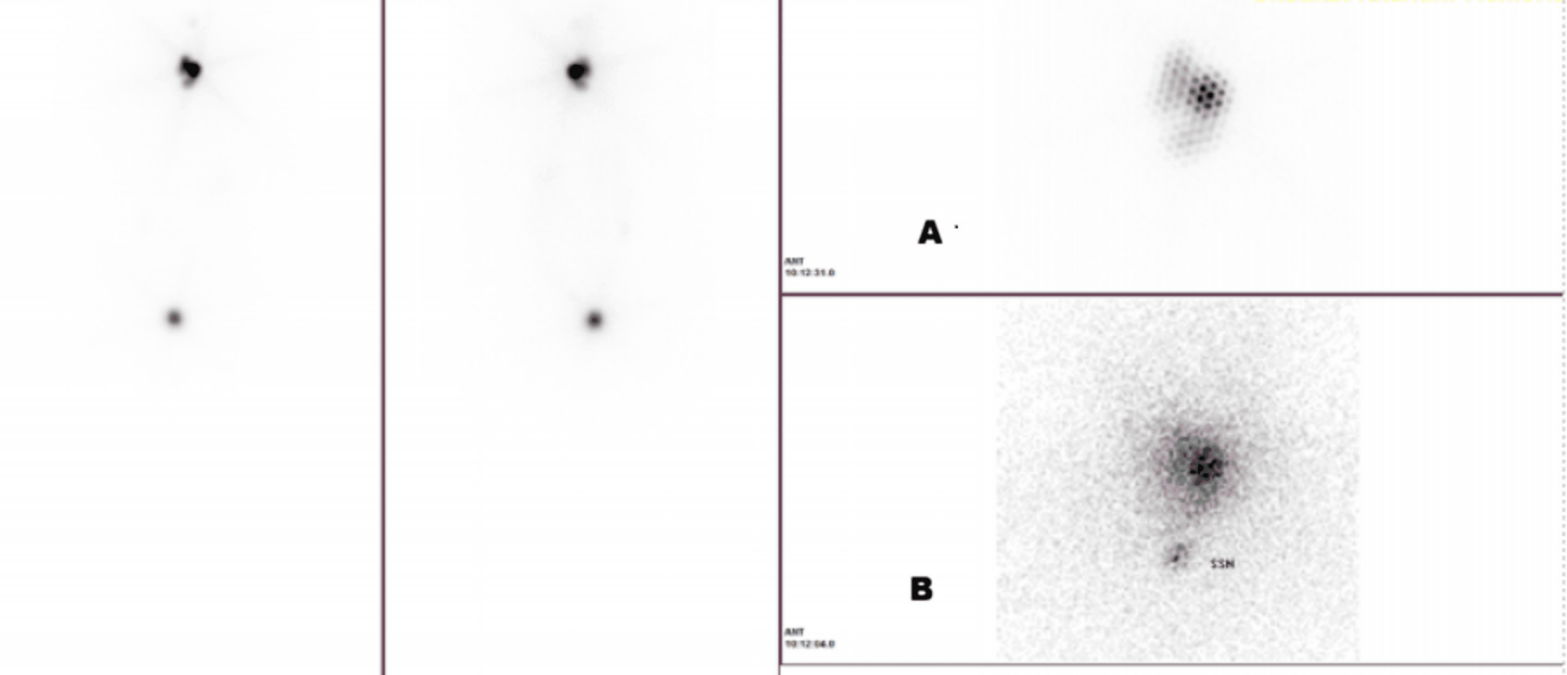
Case 2: post-radioactive iodine therapy whole body scan ((A) iodine uptake in the thyroidectomy bed and (B) iodine uptake in the adnexa)

The post-radioactive iodine therapy whole body scan showed uptake in the thyroidectomy bed and intense uptake in the adnexa (Figure 6).

This raised the suspicion of thyroid tissue in the adnexa.

**Figure 7:**
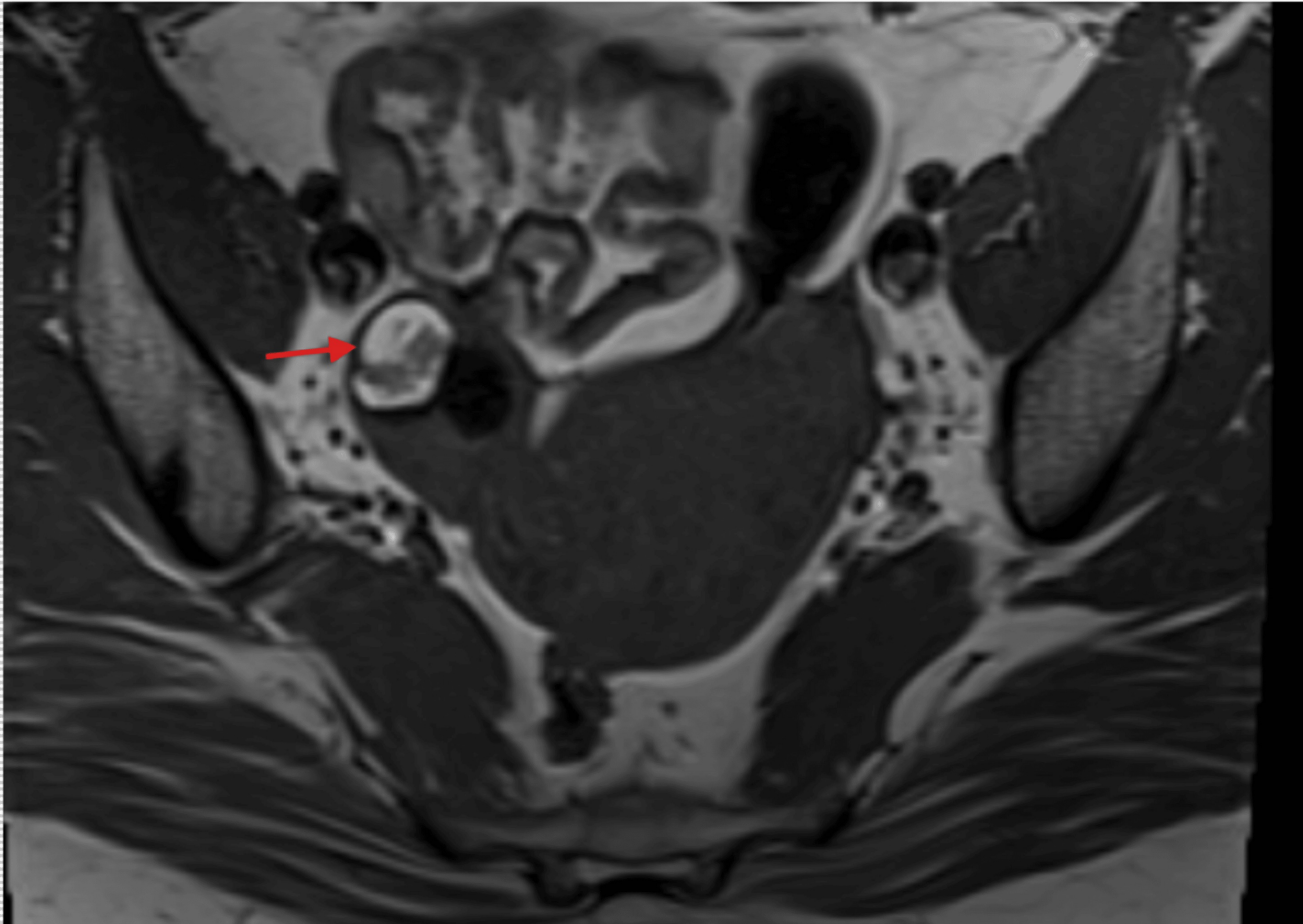
Case 2: MRI of the pelvis (red arrow: dermoid cyst in the right adnexa)

MRI of the pelvis was performed, and the report stated that there was a lesion measuring 20 × 15 mm seen in the right adnexa which appears high on T1- and T2-weighted images and shows suppression on post-contrast fat-saturated images suggestive of a fat-containing lesion likely dermoid (Figure 7).

The case was again discussed in the thyroid multidisciplinary team, and it was decided to proceed with the removal of the adnexal mass as part of thyroid cancer treatment.

She underwent "laparoscopic right-sided salpingo-oophorectomy with infracolic omentectomy, appendectomy, peritoneal wall biopsy, and washings".

**Figure 8:**
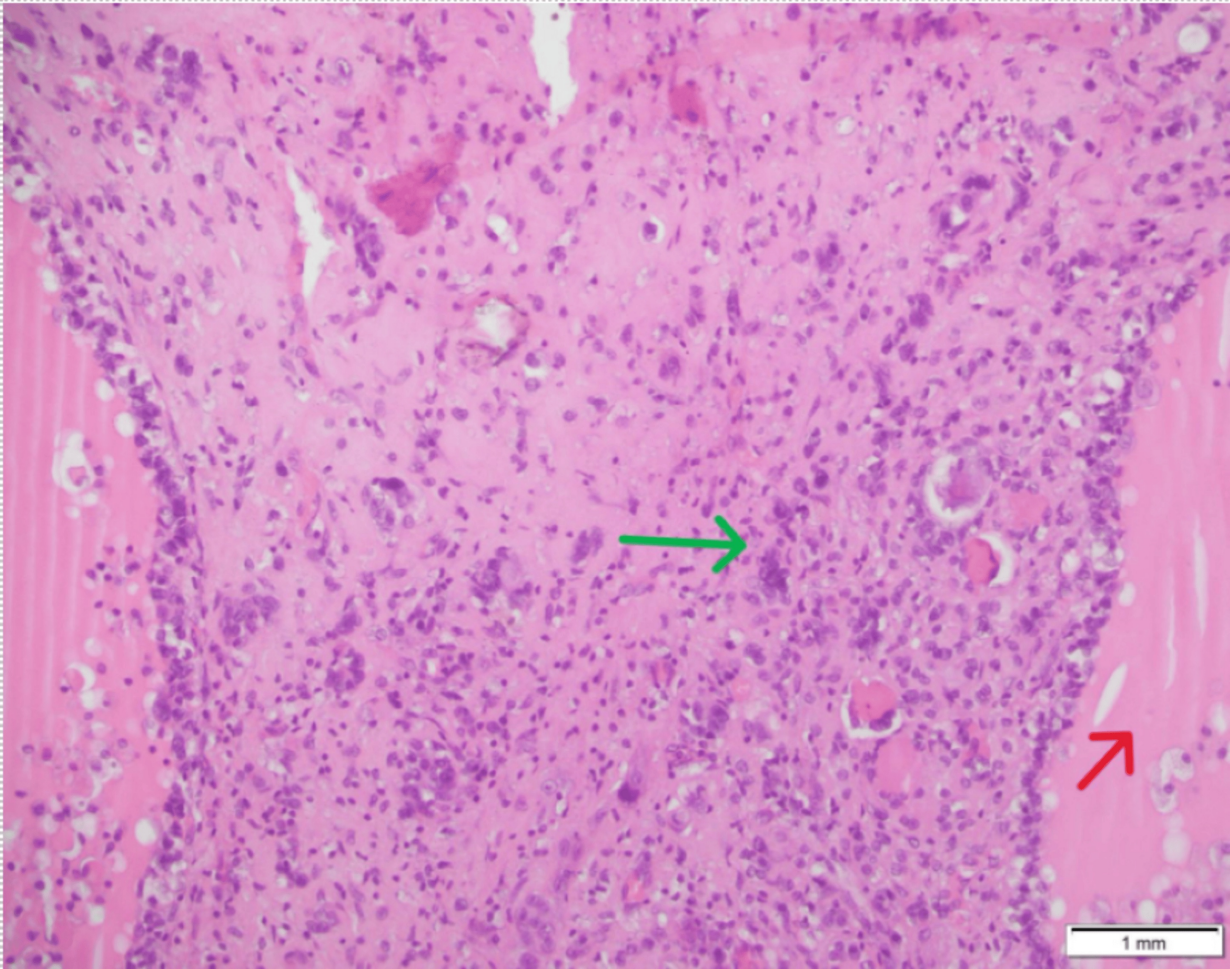
Case 2: histopathology of the ovary (red arrow: mature component of teratoma; green arrow: poorly differentiated thyroid carcinoma)

Surgical findings revealed a 3 cm right ovarian cyst (Figure 8).

This patient is currently on endocrinology follow-up and disease-free.

## Discussion

There are no established guidelines on the management of thyroid carcinoma in ovarian teratoma due to its rarity. Thyroid function tests including thyroid-stimulating hormone (TSH), free thyroxine (free T4), and free triiodothyronine (free T3) are required to establish thyroid status. Serum thyroglobulin, a glycoprotein produced by thyroid follicular cells, serves as a tumor marker in differentiated thyroid cancer [9]. It is not recommended to check thyroglobulin measurement before thyroid surgery as it can originate from benign and malignant thyroid tissue, limiting its diagnostic specificity. Following the complete removal of normal and malignant thyroid tissue, thyroglobulin is a sensitive marker that assesses residual, relapse, and metastatic disease.

These tumors are extremely rare, and there is a lack of established guidelines, and each case is treated individually with a multidisciplinary team approach comprising an endocrinologist, a gynecologist, a thyroid surgeon, and a pathologist.

Review of literature suggests that few authors are in favor of total thyroidectomy which can help in follow-up in the future with thyroglobulin tumor markers and radioactive iodine imaging. Others are inclined towards conservative management in low-risk patients [10].

In our first case, after a multidisciplinary team meeting, it was decided to proceed with total thyroidectomy, which helped us in a smooth follow-up with thyroglobulin tumor markers.

In patients with malignant thyroid tissue found in the ovary, it should always be considered whether it is a metastatic deposit from the thyroid. This question can be answered by reviewing the histopathology of ovarian tissue. In patients with thyroid cancer metastasis to the ovary, normal ovarian parenchyma can be appreciated, but in patients with the de novo development of papillary thyroid carcinoma in ovarian teratoma, the ovarian tissue is replaced by teratoma. A literature review also shows that the possibility of thyroid cancer metastasis to the ovary is less as compared to differentiated thyroid cancer development in teratoma [11,12].

In our first case, an ovarian histopathology evaluation showed the focus of thyroid carcinoma in a teratoma which was replacing the ovarian tissue.

Molecular testing can help in risk stratifying in these patients and help in surgical decision-making [13].

BRAF (point mutation in V600), ret/papillary thyroid carcinoma rearrangement, and point mutation in Harvey rat sarcoma viral oncogene homolog (HRAS) and neuroblastoma RAS viral oncogene homolog (NRAS) are reported in papillary thyroid carcinoma in ovarian teratoma. These molecular genetics help in differentiating benign from malignant lesions [14].

BRAF mutation is found in approximately 45% of sporadic papillary thyroid carcinoma [15].

Synchronous presence of papillary thyroid carcinoma in ovarian teratoma and in the thyroid is rare. A few cases of synchronous papillary thyroid carcinoma in the thyroid and malignant struma ovarii have been reported in the literature.

Earlier studies identified BRAF driver mutations in malignant struma ovarii. This suggests a shared pathogenic mechanism of papillary thyroid carcinoma regardless of anatomic location. Limited availability of data on this precludes a more definitive conclusion [16].

The second case was the simultaneous presence of two separate thyroid malignancies in ovarian teratoma (i.e., poorly differentiated thyroid carcinoma) and in the thyroid gland (i.e., papillary thyroid carcinoma).

Poorly differentiated thyroid carcinoma is placed between highly differentiated papillary and follicular thyroid carcinomas, with good prognosis, and anaplastic carcinoma, with poor prognosis [17].

Turin criteria, used by pathologists to diagnose poorly differentiated thyroid carcinoma, are as follows: (1) solid/trabecular architecture, (2) absence of nuclear features of papillary thyroid carcinoma, (3) tumor necrosis, (4) mitotic index >3/10 high-power fields, and/or (5) convoluted tumor nuclei.

Different stains can help to differentiate poorly differentiated thyroid carcinoma arising in ovarian teratoma from other ovarian tumors. Poorly differentiated thyroid carcinoma is positive for thyroglobulin, thyroid transcription factor-1 (TTF-1), and paired box gene 8 (PAX-8) [18].

In our second case, the ovarian histopathology specimen was positive for thyroglobulin and TTF-1 and exhibited features of Turin criteria, leading to the diagnosis of poorly differentiated thyroid carcinoma.

There are only a few cases reported in literature, approximately 10, of poorly differentiated thyroid carcinoma in ovarian teratoma, out of which there are fewer cases of concurrent presence of separate thyroid malignancy in the thyroid gland [18].

Poorly differentiated thyroid carcinoma has many possible routes to existence; it can arise either from follicular epithelial cells or from follicular or papillary carcinoma.

In our second case, the patient had primary papillary thyroid carcinoma and poorly differentiated thyroid carcinoma in the ovarian teratoma; there is a possibility that poorly differentiated thyroid carcinoma in the ovarian teratoma might have progressed from papillary carcinoma.

Due to the lack of reported cases in the literature and their rarity, each case is treated on an individual basis with a multidisciplinary approach.

## Conclusions

The presence of thyroid cancer in ovarian teratoma has a unique diagnostic and therapeutic challenge due to its rarity. A multidisciplinary approach incorporating a clinical, histological, and imaging evaluation is mandatory for accurate diagnosis and optimal treatment outcomes. Molecular testing can help in guiding treatment.
